# Optimization of Thermoplastic Pultrusion Parameters of Jute and Glass Fiber-Reinforced Polypropylene Composite

**DOI:** 10.3390/polym16010083

**Published:** 2023-12-27

**Authors:** Ponlapath Tipboonsri, Supaaek Pramoonmak, Putinun Uawongsuwan, Anin Memon

**Affiliations:** 1 Department of Industrial Engineering, Faculty of Engineering, Rajamangala University of Technology Thanyaburi (RMUTT), Thanyaburi, Phathum Thani 12110, Thailand; ponlapath_t@mail.rmutt.ac.th (P.T.); supaaek.p@en.rmutt.ac.th (S.P.); 2 Department of Materials and Production Technology Engineering, King Mongkut’s University of Technology North Bangkok (KMUTNB), Bangkok 10800, Thailand; putinun.u@eng.kmutnb.ac.th

**Keywords:** thermoplastic pultrusion, unidirectional hybrid composite, jute/glass fiber reinforced polypropylene

## Abstract

Thermoplastic pultrusion is a suitable process for fabricating continuous unidirectional thermoplastics with a uniform cross-section, high mechanical properties due to continuous fiber reinforcement, low cost, and suitability for mass production. In this paper, jute and glass fibers were reinforced with a polypropylene matrix and fabricated using the thermoplastic pultrusion process. The volumetric fraction of the composite was designed by controlling the filling ratio of the reinforcing fiber and matrix. The effects of molding parameters were investigated, such as pulling speed and molding temperature, on the mechanical properties and microstructure of the final rectangular profile composite. The pulling speed and molding temperature varied from 40 to 140 mm/min and 190 to 220 °C, respectively. The results showed that an increase in molding temperature initially led to an increase in mechanical properties, up to a certain point. Beyond that point, they started to decrease. The resin can be easily impregnated into the fiber due to the low viscosity of thermoplastic at high temperatures, resulting in increased mechanical properties. However, the increase in molding temperature also led to a rise in void content due to moisture in jute fiber, resulting in decreased mechanical properties at 210 °C. Meanwhile, un-impregnation decreased with the increase in molding temperature, and the jute fiber began to degrade at high temperatures. In the next step, with varying pulling speed, the mechanical properties decreased as the pulling speed increased, with a corresponding increase in void content and un-impregnation. This effect occurred because the resin had a shorter time to impregnate the fiber at a higher pulling speed. The decrease in mechanical properties was influenced by the increase in void content and un-impregnation, as the jute fiber degraded at higher temperatures.

## 1. Introduction

Currently, environmentally friendly products, known as ‘green products’, have continuously gained interest due to the global trend of environmental conservation, driven by campaigns promoting these green products. Composite-based green products, developed with a focus on sustainability, have garnered attention for their recycling, biodegradability, ease of disposal, and non-toxic properties, aligning with the goals of sustainable development [1]. Composite materials typically involve combining at least two materials with differing properties, comprising a matrix and reinforcement [2]. Currently, natural fibers are widely used as reinforcements due to their biodegradability, low cost, good mechanical properties, low density, and their contribution to reducing the reliance on synthetic fibers, aligning with the concept of green composites [3,4,5].

The fabrication of composites involves various processes, with popular methods including hand lay-up, resin transfer molding, hot plate compression molding, pultrusion, and filament winding [6]. The pultrusion process represents a form of continuous composite fabrication, characterized by a uniform cross-section, high mechanical properties due to continuous reinforcement fibers, cost-effectiveness in terms of equipment, and the potential for mass production [7]. Typically, the pultrusion process utilizes thermoset resin as the matrix for the composite. In this process, thermosetting resins impregnate continuous fibers before being pulled through a hot die for curing [8]. More recently, the pultrusion process has evolved by incorporating thermoplastic as the matrix, known as the thermoplastic pultrusion process. In this variant, reinforcement fibers and thermoplastic matrix fibers are pulled through a hot die, with the thermoplastic fibers melting and impregnating the reinforced fibers before taking shape through cooling [9]. With a growing focus on environmentally friendly products, ongoing research into thermoplastic pultrusion is of interest. The advantage of thermoplastic pultrusion lies in its use of thermoplastic materials, known for their recycling properties and their ability to contribute to pollution control [6]. Thermoplastic composites exhibit high ductility, fracture toughness, impact resistance, recyclability, and rapid processing. Meanwhile, thermosetting composites have low ductility, fracture toughness, and impact resistance, and are non-recyclable [10,11,12].

Jute fibers offer an alternative reinforcement material for enhancing composite materials due to their lightweight, affordability, environmental friendliness, and local availability as a continuous fiber [6,13,14]; they are suitable for the thermoplastic pultrusion process. Additionally, jute fibers contain a high amount of cellulose, approximately 64.40%, making them suitable for reinforcement [5]. While natural fibers are highly eco-friendly as reinforcement in composite materials, their mechanical properties are typically low. To address this limitation and boost the mechanical properties of composites, a hybrid composite approach is employed, combining two or more types of reinforcement. This serves as an alternative for enhancing the mechanical properties of composites. Glass fibers are also widely utilized in the plastic composite industries, prized for their high strength, cost-effectiveness, high melting temperature, and continuous fiber shape [15].

**Figure 1:**
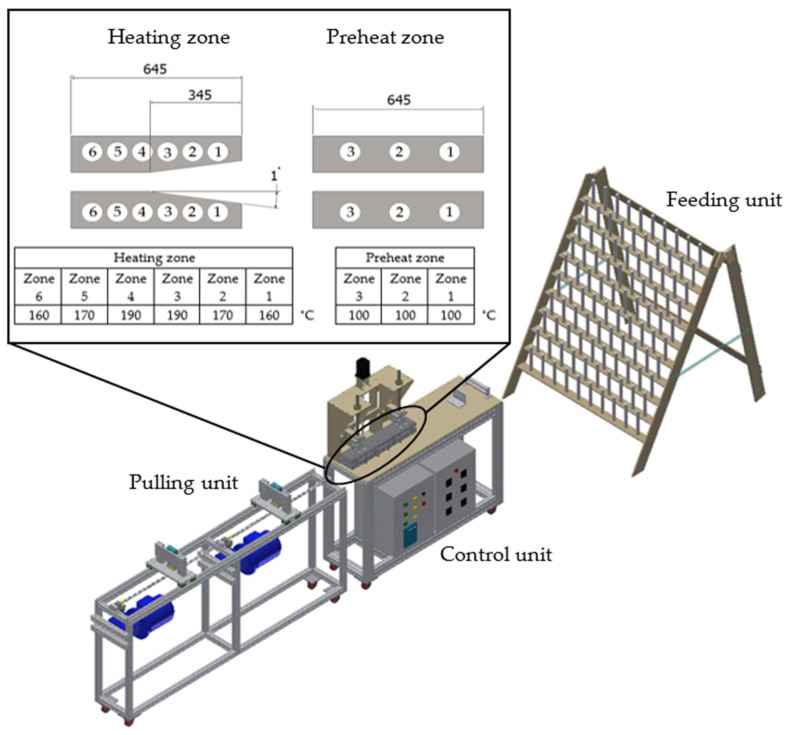
Schematic of thermoplastic pultrusion process and heating position of die.

Polypropylene (PP) is a widely popular thermoplastic used in various industries, including automotive, food, and packaging, among others [16,17]. PP exhibits low density, low processing temperature, low water absorption, and recyclability [17]. Currently, glass fiber-reinforced polypropylene (GFRPP) is utilized to enhance the mechanical properties of various products, including wind turbine blades [18]. GFRPP has been studied in many processes to determine optimal forming conditions, such as injection molding, compression molding, pultrusion molding, and 3D printing [19,20,21]. Moreover, PP exhibits good mechanical properties and benefits from a relatively low molding temperature that does not lead to the degradation of natural fibers [6]. In the existing literature, numerous studies have explored the use of jute/glass reinforcement with thermoset resins. However, it has not been extensively applied with thermoplastic materials [22,23]. This research focuses on the use of jute fiber and glass fiber as hybrid reinforcement materials with a polypropylene matrix. In the experiment, molding temperature and pulling speed were defined as parameters, and the final profile was examined to assess the mechanical properties and microstructure. In the initial phase, the hybrid composites were fabricated and investigated to determine the optimal molding temperature. Subsequently, in the next step, the pulling speed was adjusted, as illustrated in Figure 1, depicting the thermoplastic pultrusion process used in this experiment. This work provides data on the effects of thermoplastic pultrusion parameters on the mechanical properties, methodology, and processing window of hybrid composites. The jute fiber/glass fiber-reinforced polypropylene produced through thermoplastic pultrusion results in a unidirectional and continuous composite with high mechanical properties. Additionally, the utilization of jute fibers in composite materials for future green products aligns with the goal of sustainable use of renewable and environmentally friendly materials, as well as cost reduction, given that jute is obtained from a natural source.

## 2. Materials and Methods

### 2.1. Materials

Jute yarn and glass fiber (GF) were used in combination with polypropylene (PP). The primary reinforcing fiber selected was jute yarn, which had a tex of 840 and a density of 1.440 g/cm^3^, purchased from Hiab Seng Hen Co., Ltd., Bangkok, Thailand. This type of fiber has a degradation temperature of approximately 240 °C. A secondary reinforcing fiber, glass fiber (Direct roving EDR17-1200-386 China Jushi Co., Ltd., Tongxiang, China.) with a tex of 1200 and a density of 2.620 g/cm^3^, was also incorporated. The matrix fiber employed in the continuous composite was polypropylene yarn, with a tex of 133 and a density of 0.946 g/cm^3^, purchased from Praditkorn Co., Ltd., Bangkok, Thailand. The melting temperature of this matrix fiber was determined to be 178.8 °C through the analysis of results from a differential scanning calorimetry (DSC) test. The volume fraction of fiber and matrix was determined by adjusting the filling ratio [6,24], as described in Equation (1).

(1) $$\mathrm{Filling}\;\mathrm{ratio}=\frac{Cross-section\;area\;of\;materials}{Cross-section\;area\;of\;the\;molding\;die}\times 100$$

### 2.2. Molding Parameter

**Figure 2:**
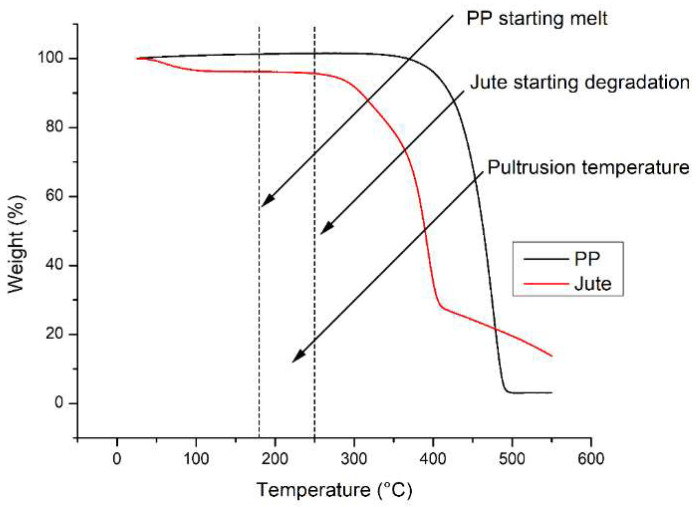
TGA results of jute fiber and pp fiber.

**Figure 3:**
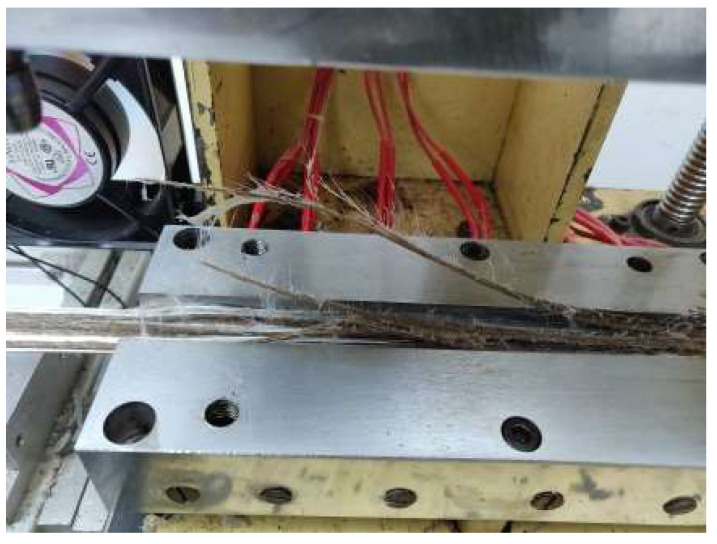
Failure of jute fiber at a temperature over 220 °C.

**Figure 4:**
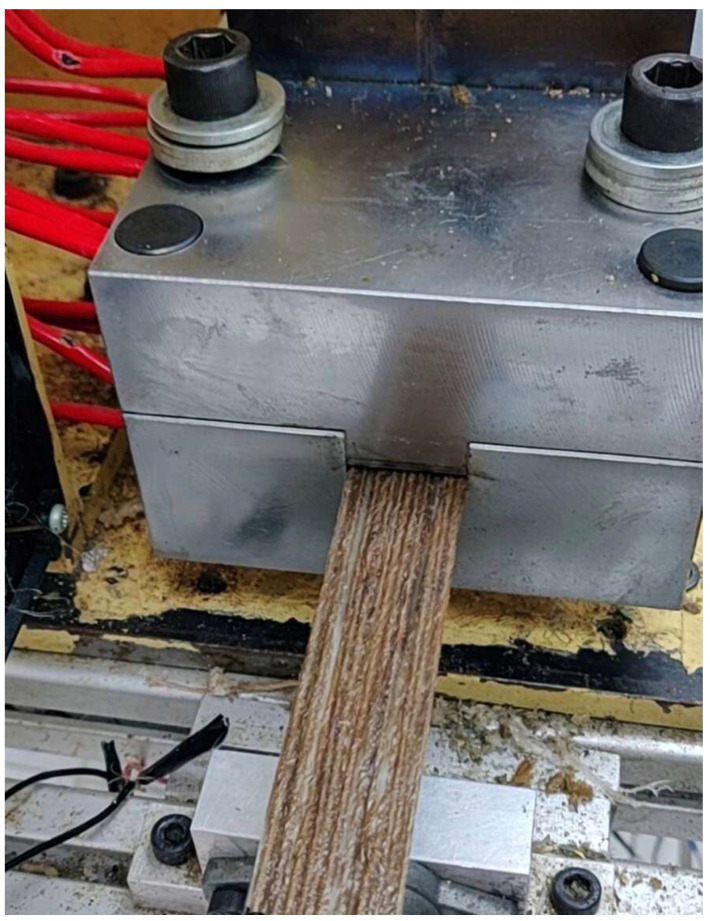
Successfully pultruded specimen of jute/glass polypropylene hybrid composite.

This study evaluated molding parameters for jute/glass fiber reinforcement with PP using the thermoplastic pultrusion process. The thermoplastic pultrusion machine is a lab-scale apparatus equipped with a heating die and a preheat die. Each die has a length of 645 mm, a constant cross-section length of 300 mm, and a taper length of 345 mm with a 1-degree radius. The cross-section of the die has a width of 25 mm and a thickness of 3 mm as shown in Figure 1. The molding temperatures and pulling speeds are detailed in Table 1. This study investigated the effect of molding temperatures on mechanical properties and impregnated quality. After selecting the optimal molding temperature, the study progressed to assess how different pulling speeds affected the mechanical properties of the composites. The volume fraction of continuous composites was designed as presented in Table 2. The filling ratio determined the intermediate material before molding. Typically, in the thermoplastic pultrusion process, the filling ratio is configured to exceed 100% of the cross-sectional area of the die [6,25]. This design enables an excess of thermoplastic fiber, beyond 100%, to undergo impregnation or backflow phenomena at the taper zone of the die, thereby enhancing impregnation quality. Therefore, the filling ratio of the specimen consisting of PP was 68.45%, and for jute fiber was 23.06% and 9.01%. However, the volume fraction of the specimen consisting of PP was 67.93%, and for jute fiber was 23.06% and 9.01%. The volume fraction of the reinforced fibers was 32.07%, as designed according to the literature. An increase in the volume fraction of reinforced fibers can make it difficult for the resin to impregnate the fibers for the thermoplastic pultrusion process. Pulling speed was measured based on the frequency of the pulling motor revolutions, resulting in values of 40, 100, and 140 mm/min at 4, 6, and 8 Hz, respectively. The die temperature system for this experiment is illustrated in Figure 1, with heating zones 1 and 2 of the die considered to be associated with the melting point of PP fibers. It was essential to avoid the melting of PP fibers in zones 1 and 2 to prevent fiber breakage before reaching the die. Therefore, the die setup for zones 1 and 2 was configured at 160 and 170 °C, temperatures lower than the melting temperature of 178.8 °C as determined by DSC results. The die temperature system for zones 3 and 4 (Impregnation zone) was designed based on the results of Thermal Gravimetric Analysis (TGA) of jute fiber and PP fiber, as depicted in Figure 2. The TGA results indicated that the degradation temperature of jute fibers was approximately 240 °C and the degradation temperature of PP fibers around 290 °C. The melting temperature of PP was 180 °C. Consequently, the suitable range for molding temperature fell within the bracket of 180 °C to 240 °C. However, despite the degradation temperatures of PP fibers and jute fibers being 290 °C and 240 °C, respectively, the molding temperature could not be set above 220 °C, as it would approach the degradation temperature of jute fiber. The failure of jute fiber at temperatures exceeding 220 °C was confirmed in Figure 3. Consequently, the molding temperatures were established at 190 °C, 200 °C, 210 °C, and 220 °C. Figure 4 displays successfully pultruded specimens of the jute/glass polypropylene hybrid composite. In addition, the design of molding temperatures took into account the pulling speeds. For pulling speeds of 40, 100, and 140 mm/min, intermediate materials were pulled through the die in approximately 16.16, 6.45, and 4.61 min, respectively. In Figure 1, the die temperature system of this experiment is illustrated, with variations occurring only in zones 3 and 4, while the preheat zones were set at 100 °C. The selection of pulling speeds was based on the impregnation theory outlined in the literature [6,7,17,22,24] which suggests that resin can adequately impregnate the materials over an extended period at lower pulling speeds. As a result, the preliminary pulling speed was set at the lowest value compatible with pultrusion techniques.

### 2.3. Thermal Gravimetric Analysis (TGA)

Thermal stabilities of jute fiber and polypropylene (PP) were investigated using Thermogravimetric Analysis (TGA) to determine their degradation temperatures. The TGA (Perkin-Elmer model 4000, Waltham, MA, USA) was programmed to record data from 25 to 550 °C at a heating rate of 10 °C/min. The specimens were heated in a nitrogen atmosphere.

### 2.4. Differential Scanning Calorimetry (DSC)

DSC (Netzsch model DSC 200 f3, Selb, Germany) was used to investigate the melting point of polypropylene (PP) for the design of the molding temperature of the composite. The DSC conditions were set with a heating rate/cooling rate of 10 °C/min and a recorded temperature range from 25 to 300 °C.

### 2.5. Mechanical Test

Tensile and flexural tests were carried out to investigate the mechanical properties. The dimensions of the tensile specimens were 25 × 250 × 4 mm in accordance with ASTM D3039 [26]. These specimens had a gauge length of 150 mm, with 50 mm clamped at each end. Tensile tests were conducted using a Hounsfield Universal Testing machine, with a head speed set at 1 mm/min, which is lower than the head speed specified in ASTM D3039. This is because the specimens failed before 1 min, which is less than the recommended time range of 1–10 min specified in the standard.

The flexural test was performed using a three-point bending test following ASTM D790 [27]. The flexural specimens had dimensions of 25 × 100 × 4 mm. Flexural tests were conducted on a Hounsfield Universal Testing machine, with a head speed set at 2.16 mm/min and a span length of 72 mm. A head speed can be calculated by Equation (2), where R = rate of crosshead (mm/min), L = support span (mm), d = depth of beam (mm), and Z = rate of straining of the outer fiber (Z shall be equal to 0.01).

(2) $$R=\frac{{ZL}^{2}}{6d}$$

### 2.6. Microstructure Analysis

**Figure 5:**
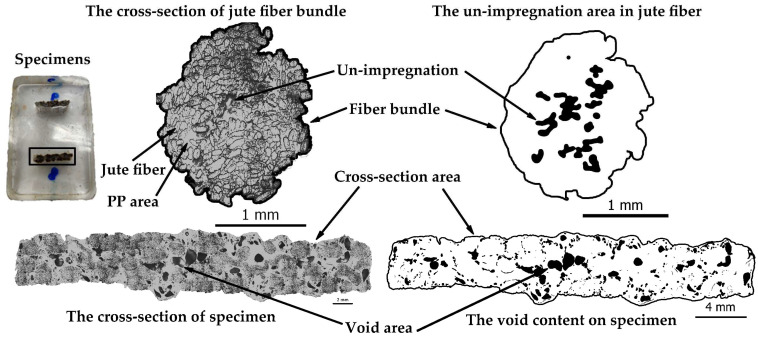
Analysis of the difference between void content and un-impregnation.

For the preparation of specimens for microstructure analysis, resin casting was employed. The specimens were subsequently polished using a polishing machine and then examined under a microscope. The microstructure analysis was performed using an Olympus microscope, with samples examined at 10 times magnification. This analysis primarily concentrated on void and un-impregnation assessment. Un-impregnation was determined by comparing the space area within the fiber bundle to the fiber bundle area, as illustrated in Figure 5, where the black region within the fiber bundle represents un-impregnation. Void content, on the other hand, was calculated by measuring the void area in relation to the cross-section area, as also indicated in Figure 5, where the black region on the specimen’s cross-section denotes void content.

## 3. Results and Discussions

In this experiment, the initial set of specimens entailed altering the molding temperatures to identify the most suitable temperature for subsequent investigations. The varying molding temperatures were adjusted based on Table 1, focusing exclusively on zones 3 and 4. After selecting the optimal molding temperature, this study progressed to assess how different pulling speeds affected the mechanical properties of the composites.

### 3.1. The Effect of Molding Temperature Parameter

**Figure 6:**
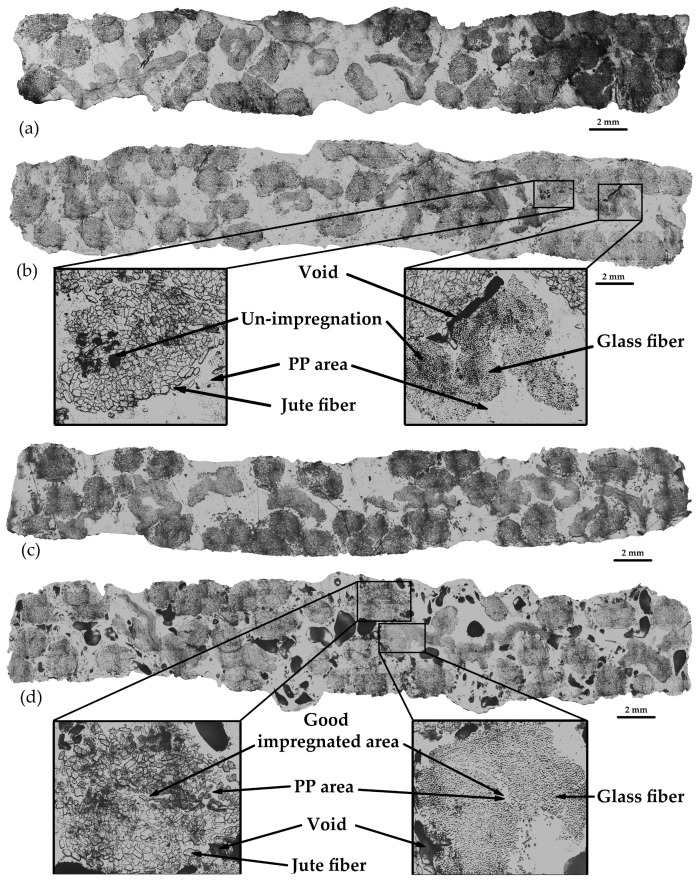
Cross-section of differential molding temperatures: (**a**) specimen at 190 °C, (**b**) specimen at 200 °C, (**c**) specimen at 210 °C, and (**d**) specimen at 220 °C.

**Figure 7:**
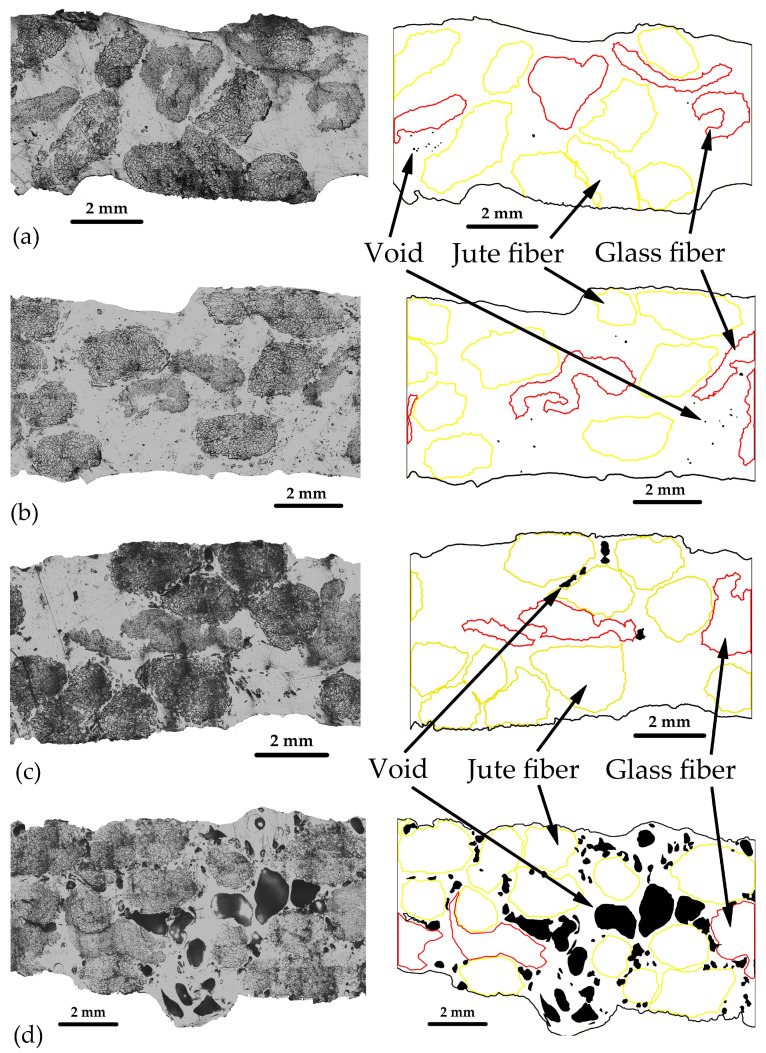
Cross-section observation of specimens: (**a**) specimen at 190 °C, (**b**) specimen at 200 °C, (**c**) specimen at 210 °C, and (**d**) specimen at 220 °C.

**Figure 8:**
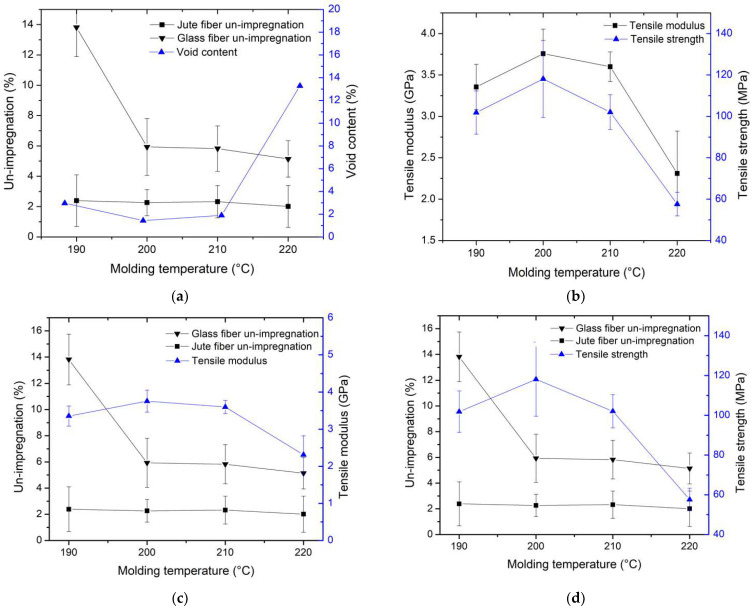
Relationship between (**a**) un-impregnation, void content, and molding temperature, (**b**) tensile modulus, tensile strength, and molding temperature, (**c**) un-impregnation, tensile modulus, and molding temperature, and (**d**) un-impregnation, tensile strength, and molding temperature.

Figure 6 presents the microstructure of composite specimens at various molding temperatures. The findings reveal that the void content exhibited a noticeable increase at 220 °C, with a starting point for increased void content at 210 °C, as depicted in Figure 6d. Additionally, Figure 7 provides magnified cross-section areas that reveal the positions of jute fiber, glass fiber, and void content. It is clear that higher molding temperatures led to an increase in void content, while the un-impregnation of resin decreased as the molding temperature rose. The presence of void content in the specimens may result from trapped moisture within the jute fiber, which is released at higher molding temperatures [5]. From the microstructure results, the void content and un-impregnation analysis with mechanical properties are shown in Figure 8a–d. The higher temperature affected the speed of moisture evaporation from the jute fiber.

The results regarding the correlation between un-impregnation, void content, and molding temperature can be found in Figure 8a, whereas the relationship between molding temperature and tensile modulus, as well as tensile strength, is depicted in Figure 8b. In the un-impregnation results, there was a clear tendency for un-impregnation to decrease with an increase in molding temperature. Resin exhibited a greater ease of impregnation into the jute fiber compared to the glass fiber, primarily due to the jute fiber having more filament space within each bundle than the glass fiber. Notably, there was a significant issue with resin un-impregnation at a molding temperature of 190 °C for the glass fiber, with a rate as high as 13.81%. As temperatures increased, resin impregnation into the fiber became more effective compared to lower temperatures. This indicates that the resin viscosity decreases as the molding temperature increases, making it easier for the resin to impregnate. Effective resin impregnation into the fibers is crucial for enhancing mechanical properties. The results of void contents, presented in Figure 8a, demonstrate that void content began to increase at 210 °C, with the highest void content at 220 °C being approximately 13.28% and the lowest void content at 200 °C being about 1.44%. The presence of void content in the composite results from moisture in the jute fiber [5]. In this case, voids appeared when the molding temperature exceeded 210 °C, possibly due to interactions between the cellulose in the jute and water molecules [28].

The presence of void content directly affects the mechanical properties of the composites [29]. Both tensile modulus and tensile strength exhibited a decline as void content increased. These findings reveal that tensile modulus increased as the molding temperature escalated up to 200 °C, but thereafter, it began to decline once the temperature surpassed 210 °C. The highest tensile modulus value was 3.76 GPa at a molding temperature of 200 °C. Tensile strength followed a similar trend, with the highest value of approximately 118.13 MPa occurring at a molding temperature of 200 °C. This effect can be attributed to the decreased viscosity of the resin at higher molding temperatures, facilitating its easier impregnation into the reinforcing fibers [6,30,31]. Typically, resin can easily impregnate when it exhibits low viscosity or when molding temperatures are high [6,32]. However, in this case, at 220 °C, the jute fiber experienced high un-impregnation, possibly due to the presence of voids within the jute bundle, which further contributes to increased un-impregnation. Moreover, the decrease in tensile strength and modulus at 210 °C is attributed to the degradation of the jute fiber, which initiates decomposition at 240 °C [32,33].

Figure 8c illustrates the relationship between un-impregnation, molding temperature, and tensile modulus, while Figure 8d presents the relationship between un-impregnation, tensile strength, and molding temperature. These results demonstrate the impact of un-impregnation on tensile modulus and tensile strength. Un-impregnation of the resin in the fibers decreased with an increase in molding temperature. Lower un-impregnation resulted in higher tensile modulus and tensile strength. However, even at 220 °C, where un-impregnation was low, tensile modulus and tensile strength remained low due to other factors, including void content and the degradation of the jute fiber.

**Figure 9:**
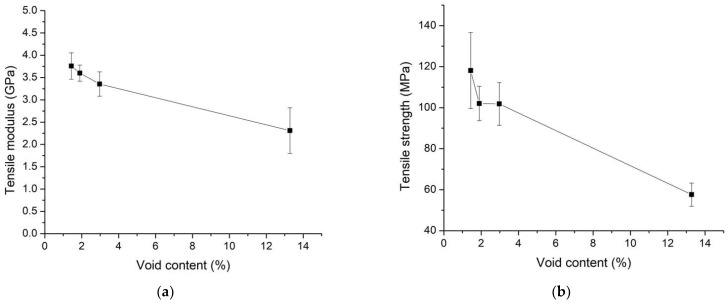
Relationship between (**a**) tensile modulus and void content, and (**b**) tensile strength and void content.

The void content of the specimens directly influenced their mechanical properties, with mechanical properties decreasing as void content increased. The void content at various molding temperatures was measured at 1.44%, 1.9%, 2.97%, and 13.28%, respectively. The relationships between (a) tensile modulus and void content and (b) tensile strength and void content are depicted in Figure 9. It is evident that tensile strength decreased with an increase in void content, consistent with findings in the literature [29,33]. The presence of void content created spaces within the specimen, leading to a decrease in tensile strength and tensile modulus. These voids originated from moisture within the jute fiber.

**Figure 10:**
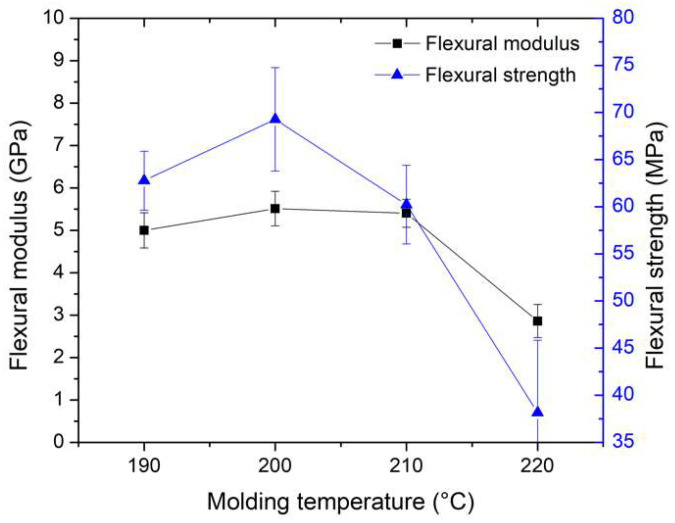
Relationship between flexural modulus, flexural strength, and molding temperature.

The results, as depicted in Figure 10, illustrate the connection between molding temperature, flexural modulus, and flexural strength, exhibiting a pattern akin to the tensile properties. A rise in the molding temperature led to an increase in both flexural modulus and strength, up to 210 °C, beyond which they showed a decline. The peak values for flexural modulus and strength, reaching 5.51 GPa and 69.27 MPa, respectively, were observed at a molding temperature of 200 °C.

Even though resin impregnation was good, the mechanical properties were not always favorable. This suggests that the mechanical properties at a molding temperature of 220 °C decreased, as they were also influenced by void content and the degradable temperature of the jute fiber. The results show that the highest void content at 220 °C of molding temperature had a significant impact on the previously low mechanical properties. Additionally, at 220 °C of molding temperature, the jute fibers began to degrade. Based on the microstructure analysis and the discussion of mechanical properties, a molding temperature of 200 °C was selected as the optimal temperature for further investigation into the influence of pulling speed.

**Figure 11:**
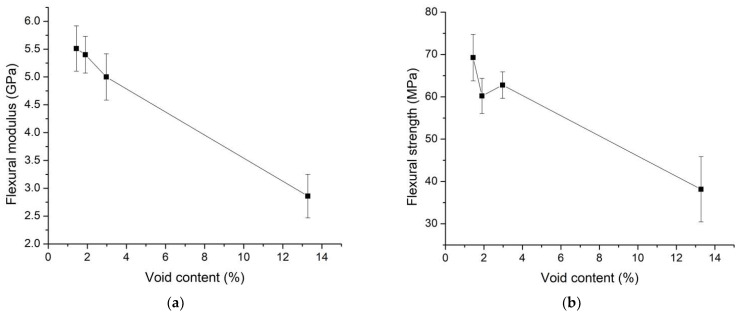
Relationship between (**a**) flexural modulus and void content, and (**b**) flexural strength and void content.

The relationships between flexural modulus, flexural strength, void content, varied molding temperature, and pulling speed are presented in Figure 11. The results indicated that flexural strength and flexural modulus exhibited a trend similar to that of tensile strength and tensile modulus. They both decrease with an increase in void content. Consequently, the presence of void content in the specimens contributed to a decrease in mechanical properties. The slight increase in flexural strength from the second point to the third point in Figure 11b is attributed to the good impregnation quality of resin in the fiber at that point. Therefore, the fraction of impregnation quality and void content has an effect on mechanical properties.

### 3.2. The Effect of Pulling Speed Parameter

After a study of the molding temperature parameter, the molding temperature was selected at 200 °C for the study of pulling speed parameters. The pulling speed is one parameter that is important in the pultrusion process. Normally, pulling speed parameters can affect resin impregnation [25,34,35].

**Figure 12:**
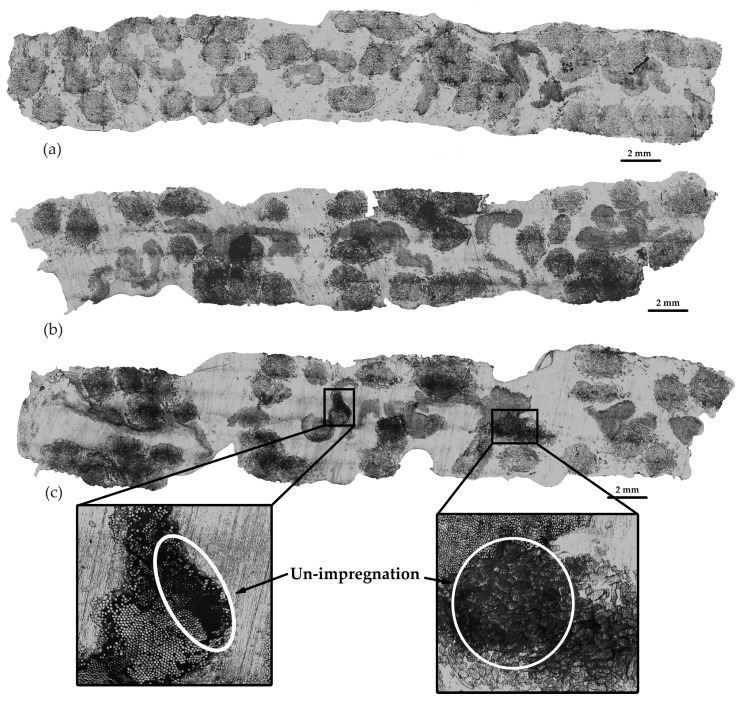
Cross-section of different pulling speeds: (**a**) specimen at 40 mm/min, (**b**) specimen at 100 mm/min, and (**c**) specimen at 140 mm/min. There are differences in the length of cross-section figures because of different pulling speeds. The molding temperature was selected at 200 °C for the study of the effect of pulling speed parameters.

Figure 12 presents the analysis of the microstructure of composite specimens at various pulling speeds, ranging from 40 to 140 mm/min, through microscopic examination. The findings revealed that un-impregnation grew with an escalation in pulling speed, as seen in Figure 12c. Following this, an assessment of void content and un-impregnation was carried out.

**Figure 13:**
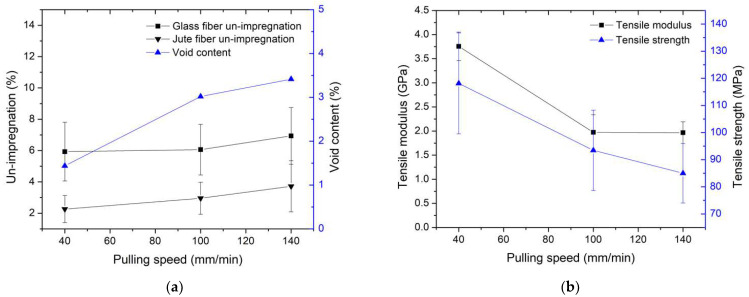
Relationship between (**a**) un-impregnation, void content, and pulling speed, and (**b**) tensile modulus, tensile strength, and pulling speed. The molding temperature was selected at 200 °C for the study of the effect of pulling speed parameters.

The results regarding the relationship between un-impregnation, void content, and pulling speed are presented in Figure 12a, while the relationship between tensile modulus, tensile strength, and pulling speed is depicted in Figure 12b. Similar to the un-impregnation results at different molding temperatures discussed previously, it was observed that the un-impregnation of glass fiber was higher than that of jute fiber. This difference can be attributed to the reduced space between the filaments in glass fiber compared to jute fiber. As a result, the resin can impregnate the glass fiber bundle to a lesser extent than the jute fiber bundle, as shown in Figure 12c in the magnified area, illustrating un-impregnation between glass fiber and jute fiber. In the sections involving different pulling speeds, un-impregnation increased with an increase in pulling speeds [24,25,35] because the resin can impregnate over a longer duration at lower pulling speeds. Consequently, higher pulling speeds allow for shorter impregnation times. This explains why the un-impregnation value can be used to confirm the decrease in mechanical properties at higher pulling speeds, in accordance with previous literature. The results also showed an increase in void contents with higher pulling speeds. However, these variations in void content were relatively small, ranging from about 1% to 2%, with the lowest void value observed at 40 mm/min pulling speed, at approximately 1.44% of void content. These minimal differences had limited effects on the mechanical properties. The tensile modulus decreased with an increase in pulling speed because specimens remained in the mold for a longer time, allowing the resin to impregnate for an extended period. The results indicated a tensile modulus of 3.76 GPa at a pulling speed of 40 mm/min. Tensile strength in Figure 13b exhibited a similar trend to the tensile modulus, with the highest tensile strength value of approximately 118.13 MPa observed at a pulling speed of 40 mm/min. These results align with existing literature [25,35].

**Figure 14:**
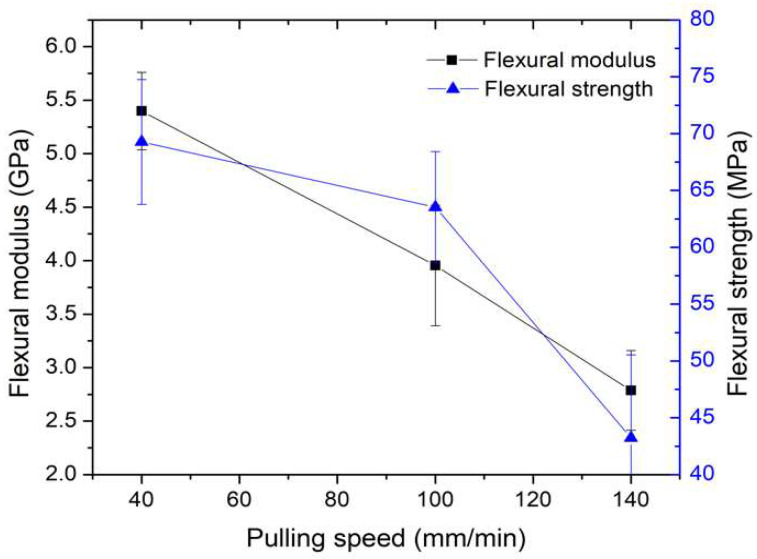
Relationship between flexural modulus, flexural strength, and pulling speed. The molding temperature was selected at 200 °C for the study of the effect of pulling speed parameters.

The results regarding the relationship between flexural modulus, flexural strength, and pulling speed are presented in Figure 14, illustrating a decrease in flexural strength and modulus with an increase in pulling speeds. Similar to the trends observed with tensile modulus and strength, these specimens experienced an increase in pulling speeds, resulting in less impregnation time for the resin, leading to lower flexural strength and modulus. Notably, the flexural strength and flexural modulus reached their highest values at a pulling speed of 40 mm/min, measuring 69.27 MPa and 5.51 GPa, respectively. As a result, a pulling speed of 40 mm/min yielded the highest mechanical properties in terms of tensile strength, tensile modulus, flexural strength, and flexural modulus.

**Figure 15:**
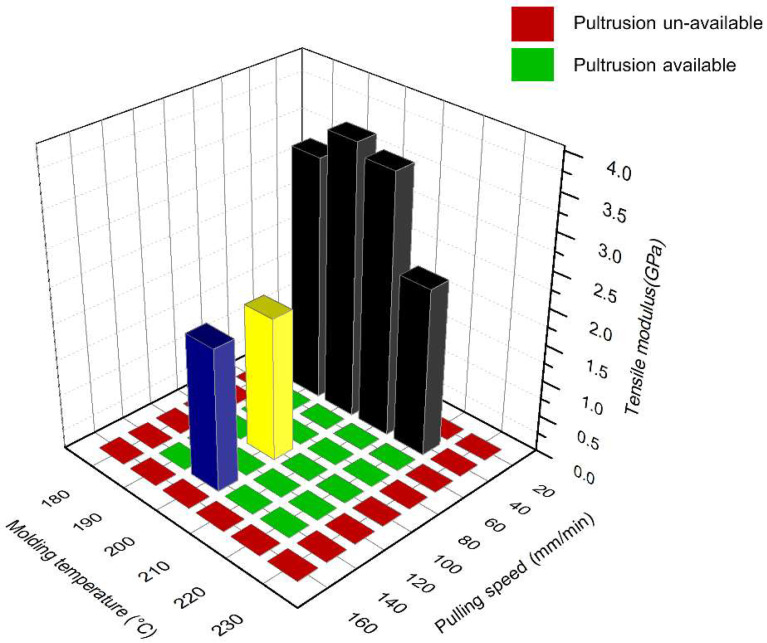
Window processing of jute/glass fiber reinforced with PP composite by thermoplastic pultrusion.

For the analysis of the results, window processing can be defined based on molding temperature and pulling speed, as shown in Figure 15. A lower molding temperature leads to poor impregnation and high viscosity of the thermoplastic, which in turn impacts the mechanical properties, given that they rely on impregnation quality. Furthermore, the high viscosity of the thermoplastic can cause jute fiber breakage before being pulled out of the hot die. Thus, the lowest feasible molding temperature for pultrusion is 190 °C. On the other hand, a higher molding temperature has a positive effect on impregnation and lowers viscosity. However, it is important to note that jute fibers start to degrade at 220 °C of molding temperature. Therefore, the recommended molding temperature range for continuous composite pultrusion is 190 to 220 °C. Regarding pulling speed, lower pulling speeds result in better impregnation, but they can also lead to the degradation of jute fiber. Conversely, higher pulling speeds are associated with lower impregnation. The acceptable pulling speed range for composite pultrusion is 40 to 140 mm/min. The optimal parameters for jute fiber/glass fiber reinforced polypropylene by the thermoplastic pultrusion process were a molding temperature of 200 °C and a pulling speed of 40 mm/min. The resulting mechanical properties were a tensile modulus of 3.76 GPa, tensile strength of 118.13 MPa, flexural modulus of 5.51 GPa, and flexural strength of 69.27 MPa.

## 4. Conclusions

In this study, jute and glass fibers were utilized to reinforce a polypropylene (PP) matrix using the pultrusion process. The pultrusion process involved the adjustment of the molding temperature and pulling speed as key parameters. To evaluate the impact of these parameters, this study examined the mechanical properties of the jute/glass polypropylene hybrid composite, specifically focusing on tensile strength, tensile modulus, flexural strength, and flexural modulus. This study led to the following conclusions:

- The mechanical properties increased with an increase in molding temperature, but after reaching 200 °C, they started to decrease. These effects were influenced by void content, un-impregnation, and the degradation of jute fibers.
- Un-impregnation decreased with an increase in molding temperature. Resin can more easily impregnate jute fiber than glass fiber due to the larger size of the fiber bundle.
- Void content increased with higher molding temperatures due to interactions between the cellulose in the jute and water molecules.
- The microstructure image of the molding temperature shows good resin impregnation in the fiber cross-section of the specimen at high molding temperatures. Meanwhile, a higher molding temperature results in the appearance of numerous void areas.
- As pulling speed increased, mechanical properties decreased, accompanied by an increase in void content and un-impregnation.
- The microstructure image of pulling speed showed good resin impregnation in the fiber cross-section of the specimen at low pulling speed.
- The optimal thermoplastic pultrusion process was a molding temperature of 200 °C and a pulling speed of 40 mm/min. The resulting mechanical properties were a tensile modulus of 3.76 GPa, tensile strength of 118.13 MPa, flexural modulus of 5.51 GPa, and flexural strength of 69.27 MPa.
- In the thermoplastic pultrusion process of jute/glass fiber reinforced with PP, a window of processing is defined by the molding temperature and pulling speed parameters. The feasible range for molding temperatures was 190 to 220 °C, and for pulling speeds, it was 40 to 140 mm/min.

This study plays a significant role in the development of pultruded composites for jute/glass fiber reinforced with PP through thermoplastic pultrusion. It marks the initiation of continuous thermoplastic pultrusion hybrid composites, aligning with green and recycling initiatives by utilizing jute fiber from nature. This alternative method broadens the application range of continuous composite materials.

## Figures and Tables

**Table 1:** Thermoplastic pultrusion conditions.

Preform No.	Temperature (°C)	Pulling Speed (mm/min)
1	190	40
2	200	40
3	210	40
4	220	40
5	200	100
6	200	140

**Table 2:** The volume fraction of continuous composite.

VF of PP (%)	VF of Jute Fiber (%)	VF of GF (%)	Filling Ratio (%)
68.45	23.06	9.01	100.52

## Data Availability

The data presented in this study are available on request from the corresponding author.
